# Strengthening health data on a rare and heterogeneous disease: sarcoma incidence and histological subtypes in Germany

**DOI:** 10.1186/s12889-018-5131-4

**Published:** 2018-02-12

**Authors:** Meike Ressing, Eva Wardelmann, Peter Hohenberger, Jens Jakob, Bernd Kasper, Katharina Emrich, Andrea Eberle, Maria Blettner, Sylke Ruth Zeissig

**Affiliations:** 1 Cancer Registry of Rhineland-Palatinate, Große Bleiche 46, 55116 Mainz, Germany; 2 University Hospital Muenster, Gerhard-Domagk-Institute of Pathology, Albert-Schweitzer-Campus 1, Gebäude D17, 48149 Münster, Germany; 3 Division of Surgical Oncology & Thoracic Surgery, Medical Faculty Mannheim, University of Heidelberg Mannheim, Theodor-Kutzer-Ufer 1-3, 68167 Mannheim, Germany; 4 University of Heidelberg, Mannheim University Medical Center, Interdisciplinary Tumor Center (ITM), Sarcoma Unit, Theodor-Kutzer-Ufer 1-3, 68167 Mannheim, Germany; 5 Institute for Medical Biostatistics, Epidemiology and Informatics, University Medical Center, Johannes Gutenberg University Mainz, 55101 Mainz, Germany; 6 Cancer Registry Bremen, Leibniz Institute for Prevention Research and Epidemiology – BIPS, Achterstraße 30, 28359 Bremen, Germany

**Keywords:** Sarcoma, Incidence, Histology, Germany, Gastrointestinal Stromal tumours, Topography, Medical, Neoplasms

## Abstract

**Background:**

The population-based incidence of sarcoma and its histological subtypes in Germany is unknown. Up-to-date information on a disease with an incidence comparable to other cancer entities is of high public health relevance. The aim of this study was to determine this incidence and to detect significant changes in incidence trends using data from German epidemiological cancer registries.

**Methods:**

Pooled data from the German Centre for Cancer Registry Data with a primary diagnosis occurring in 2013 were used. To date, this is the latest data on cancer incidence available for Germany. All German cancer registries with sufficient completeness were included (10 out of 11), covering a population of 70.0 million people, representing 87% of the German population. All malignant sarcomas according to the RARECARE Project and the WHO classification 2002 were considered for analysis and, above all, gastrointestinal stromal tumours (GIST) of uncertain behaviour. Sensitivity analysis was performed excluding certain histologies.

**Results:**

The analysis included 3404 cases in men and 3442 cases in women diagnosed in 2013. The age adjusted sarcoma incidence (European standard) was 7.4 (men) and 6.6 (women) per 100,000 inhabitants. About 70% of sarcomas were soft tissue sarcomas, about 22% GIST, and about 9% bone sarcomas. The most common histological subtypes besides GIST were fibrosarcomas (14%) and liposarcomas (12%) in men and complex mixed and stromal neoplasms (22%), non-uterine leiomysarcomas (10%) and fibrosarcomas (9%) in women. Considering the trend for the years of diagnosis 2004 to 2013, there was a significant increase in incidence for GIST while the incidence of soft tissue sarcomas (only men) as well as of bone sarcoma stayed constant over time. As to soft tissue sarcoma in women, the incidence stayed constant up to the year 2009 and significantly decreased afterwards.

**Conclusion:**

This study is the first detailed analysis of a German-wide population-based sarcoma incidence showing results comparable to the incidence detected in the RARECARE Project.

**Electronic supplementary material:**

The online version of this article (10.1186/s12889-018-5131-4) contains supplementary material, which is available to authorized users.

## Background

Sarcomas form a heterogeneous group of neoplasia emerging from mesenchymal cells [1] and can occur at almost any site of the body. According to their origin, their morphology, and their molecular genetic changes, they are divided into many different histological categories, which are mainly classified into two main groups: soft tissue and bone sarcomas [1–3].

Sarcomas account for less than 1% of all malignant tumours worldwide and thus are rare [1, 4]. However, up-to-date information on a disease with an incidence comparable to other cancer entities like cancer of the central nervous system is of high public health relevance [4].

Most analyses of cancer incidence are site-based, e.g. according to the International Classification of Diseases (ICD) [ICD-10]. In site-based classifications, there is no separate coding for sarcomas. Sarcomas emerge from many different sites [5]. Hence, sparse information is available on the incidence of all sarcoma subtypes.

An age-standardized incidence rate (2000 US standard) of 5.0 per 100,000 for soft tissue sarcomas was found in the SEER (Surveillance, Epidemiology and End results) program in the USA (years of diagnosis 1978–2001) [5].

The project “Surveillance of rare cancer in Europe” (RARECARE) provided an estimated incidence for 64 European cancer registries for cases diagnosed between 1995 and 2002 [6] of 4.2 for soft tissue sarcomas, 0.8 for bone sarcomas, and 0.1 for GIST (age-standardized (European standard), per 100,000). Further individual European studies with similar results exist [7, 8], but, to our knowledge, there is only one hospital-based study on sarcoma incidence in Germany [9].

The aim of this study was to determine the incidence of all sarcomas and their subtypes for Germany and to detect statistical significant changes in incidence in the last 10 years. For this purpose, data from the German epidemiological cancer registries provided by the German Centre for Cancer Registry Data (ZfKD) (data call December 2015; data supply from ZfKD January 2017) were used. To date, this is the latest data on cancer incidence available for Germany.

## Methods

### Data

In Germany, 11 population-based cancer registries covering the entire country exist. At the time of the data call, diagnoses up to the year 2013 were considered as sufficiently complete to be eligible for analysis. The cancer registry of Baden-Wuerttemberg had to be excluded as it was still being established in 2013. For trend analyses considering the years of diagnosis from 2004 to 2013, Hessen and Nordrhein-Westfalen (except for the district of Muenster) had to be excluded as well as they were considered complete only after the year 2004 by the ZfKD.

All malignant sarcomas according to the RARECARE Project [6, 10] and the WHO classification [1, 3, 11] were considered for analysis (Table 1) and, above all, GIST of uncertain biological behaviour. They were classified into 16 histological groups (Table 1) according to the classifications of the ICD-O-3 [12] and the WHO 2002 [1]. The complex neoplasia group consists of tumours of uncertain differentiation according to the WHO classification 2002 [1] (complex mixed and stromal neoplasia (ICD-O-3 Morphology (ICD-O-3 M) 8930–8991), synovial-like neoplasms (9040–9044)) and the malignant glomus tumour (8711). Above this, sarcomas were grouped into three entities: soft tissue sarcomas (all sites except bone (ICD-O-3 Topography (ICD-O-3 T) C40.0-C41.9); all included histologies except GIST (ICD-O-3 M 8936)), bone sarcomas (ICD-O-3 T C40.0-C41.9; all included histologies except GIST (ICD-O-3 M 8936)) and GIST (ICD-O-3 M 8936/3, 8936/1). This is modified from the RARECARE project classification of sarcomas into four main groups (tiers) [6, 10]. In contrast, we grouped the malignant GIST (8936/3) together with the GIST of uncertain malignant potential (ICD-O-3 8936/1), as the behaviour. is often hard to define in these entities. Kaposi sarcomas were not grouped as a separate entity due to the small number of cases. However, they were considered as a separate group according to histology for the sub-analysis. Sensitivity analyses were performed excluding certain histologies according to the opinion of experts or because they are missing in the WHO classification from 2002 (see Additional file 1: Table S1).

**Table 1:** Classification of histological groups^a^

Histological group	ICD-O-3 Morphology
Sarcomas NOS	8800–8806
Fibrosarcomas	8810–8840
Liposarcomas	8850–8881
Uterine leiomysarcomas	8890–8896, ICD-O-3 Topography C53, C54
Non-uterine leiomyosarcomas	8890–8896, ICD-O-3 Topography not C53, C54
Rhabdomyosarcomas	8900–8921
Complex mixed and stromal neoplasms (ICD-O-3), Others	8711, 8930–8991, 9040–9044, 9580–9581
Phylloides tumour	9020
Angiosarcomas	9120–9175
Osteosarcomas	9180–9210
Chondosarcomas	9220–9243
Giant cell neoplasia	9250–9252
Ewing family of tumours	9260, 9364, 9365
Malignant ameloblastomas	9261, 9310
Chordomas	9370–9373
Nerve sheath tumours	9540–9571

^a^according to the third edition of the International Classification of Diseases for Oncology, Morphology (ICD-O)

To compare the distribution of histological groups between different regions of the body, soft tissue sarcomas were classified into six different sites: head and neck, limbs, trunk, thorax, abdomen, and pelvis (see Additional file 2: Table S2). We chose to classify tumours in accordance with other studies in order to achieve optimal comparability. Consequently, some codes may have been summarized into the same categories although treatment strategies show relevant differences (e.g. classification of C48 into trunk tumours).

Information on the general population numbers was taken from the German Federal Statistical Office [13]. In total, a population of 69.95 million people representing 87% of the German population (key date December 31st, 2012) were included.

### Analysis

To determine the incidence of all sarcomas and their subtypes for Germany, incidence rates were calculated as the number of cases per 100,000 inhabitants, age-standardized by the European standard population 1976. Rates were analysed by sex and by year (2004 to 2013). In addition, age-specific rates in 5-year age bands were estimated, and subgroup analyses according to the sarcoma entity, histological groups, most frequent histologies, and federal states were performed. For all analyses stratified by federal state, cumulated frequencies and rates for the years 2009 to 2013 including 95% confidence intervals (95% CI) were calculated, dividing the cumulated numbers of cases by the corresponding cumulated population under risk.

For trend analyses, the most recent ten-year-period (2004 to 2013) was considered in order to enable valid analyses. In order to detect statistically significant changes in incidence in the last 10 years, annual percentage changes (APC) in the trend of the incidence rates were calculated with the statistical software Joinpoint (Joinpoint Regression Program, Version 4.4.0.0. January 2017; Statistical Research and Applications Branch, National Cancer Institute). Joinpoint uses log-linear regression to fit models with a minimal number of joinpoints (where rates change significantly) and estimates APCs and 95% confidence intervals for each section (between joinpoints): With no joinpoint, one APC was derived, with up to one joinpoint, an AAPC (average APC) was calculated. The slope of the regression line was tested to see if it was significantly different from zero.

Other analyses were performed with the software SAS 9.4 (SAS Institute, Cary NC).

Cases with unknown or unspecific histologies had to be excluded as it is unknown whether they were sarcomas. Hence, the number of sarcomas excluded is not possible to determine.

## Results

### Descriptive epidemiology and incidence rates according to sarcoma entities

In the year 2013, 6846 sarcoma cases were diagnosed in Germany (excluding Baden-Wuerttemberg) and reported to a German cancer registry (3404 men, 3442 women, Table 2).

**Table 2:** General characteristics of sarcoma patients in Germany (except for Baden-Wuerttemberg), 2013

Characteristics	Men					Women					Ratio Men/Women
	n	%	Incidence per 100,000		Median age at diagnosis	n	%	Incidence per 100,000		Median age at diagnosis	
			Crude	Age-Stand^a^				Crude	Age-Stand^a^		
All	3404	100	10.0	7.4 (7.2–7.7)	67.4	3442	100	9.6	6.6 (6.3–6.8)	67.7	1: 1
All^b^	3363	100	9.8	7.3 (7.1–7.6)	67.4	3094	100	8.7	6.0 (5.7–6.2)	67.3	1.1: 1
Type of sarcoma											
Soft tissue sarcoma	2371	69.7	6.9	5.1 (4.9–5.3)	68.4	2556	74.3	7.1	4.8 (4.6–5.0)	68.0	1: 1.1
Soft tissue sarcoma^b^	2334	69.4	6.8	5.0 (4.8–5.3)	68.3	2214	71.6	6.2	4.2 (4.0–4.4)	67.3	1.1: 1
Bone sarcoma	299	8.8	0.9	0.8 (0.7–0.9)	49.8	267	7.8	0.7	0.7 (0.6–0.8)	54.0	1.1: 1
Bone sarcoma^b^	295	8.8	0.9	0.8 (0.7–0.9)	49.8	261	8.4	0.7	0.7 (0.6–0.8)	54.2	1.1: 1
GIST	734	21.6	2.1	1.5 (1.4–1.6)	69.5	619	18.0	1.7	1.1 (1.0–1.2)	70.2	1.2: 1
Age at diagnosis (years)											
0–14	97	2.8	0.3	–	–	68	2.0	0.2	–	–	1.4: 1
15–29	158	4.6	0.5	–	–	117	3.4	0.3	–	–	1.4: 1
30–49	434	12.7	1.3	–	–	493	14.3	1.4	–	–	1: 1.1
50–69	1182	34.7	3.5	–	–	1214	35.3	3.4	–	–	1: 1
70+	1533	45.0	4.5	–	–	1550	45.0	4.3	–	–	1: 1
Total	3404	100	10.0	7.4	67.4	3442	100	9.6	6.6	67.7	1: 1

^a^per 100,000; age-standardized according to the European standard 1976

^b^Results for sensitivity analysis (excluding certain histologies according to expert opinion or due to not being mentioned in the WHO-classification, Additional file 1: Table S1)

Soft tissue sarcomas were the most frequent entity (70% in men and 74% in women), followed by GIST (22% in men and 18% in women) and bone sarcomas (9% in men and 8% in women). There was about the same number of male cases as female ones (ratio men/women 1:1). However, the age-standardized incidence rate per 100,000 was higher in men than in women (7.4 (95% confidence interval (CI): 7.2–7.7) versus 6.6 (95% CI: 6.3–6.8). For soft tissue sarcomas, there were slightly more female than male cases (ratio men/women 1:1.1). For bone sarcomas and GIST, it was vice versa (ratio men/women 1.1:1 and 1.2:1 respectively).The age-standardized incidence rates were higher for men than for women for all entities.

Excluding certain histologies according to expert opinion or because they were not mentioned in the WHO classification 2002 did not considerably alter these results in men (Table 2). In women, the exclusion of Mullerian mixed tumours (ICD-O-3 M 8950/3) resulted in a slightly lower incidence in women.

The median age at diagnosis for all sarcomas was similar in men and women (67.4 and 67.7 respectively, Table 2). While GIST cases were about 2 years older at the time of diagnosis than all other sarcoma patients, bone sarcoma patients were much younger (49.8 (men) and 54.0 years (women) respectively).

**Fig. 1:**
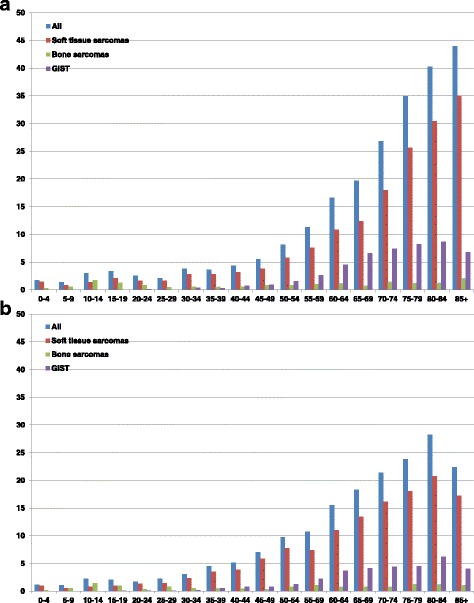
Age-specific incidence of sarcomas (per 100,000, according to the European standard 1976), year of diagnosis 2013. **a** Men; **b** Women

For all sarcomas combined, there were more male cases than female ones in the two youngest age groups (Table 2). Above the age of 30, the ratio was reversed or balanced. The incidence steadily increased with age. Regarding 5-year age groups stratified by sarcoma entity (Fig. 1), it appeared that for all entities, the incidence decreased again at ages above 85 (except for soft tissue and bone sarcomas sarcomas in men). As well, the age distribution differed for bone sarcomas, showing a first peak at ages 10 to 19 and a second peak at ages above 85 for men and of 75 to 79 for women (Fig. 1).

### Subanalysis according to histological groups

The five most important histological groups were fibrosarcoma, liposarcoma, complex neoplasia, non-uterine leiomyosarcoma, and GIST, altogether representing more than half of all sarcomas (60% in men and 65% in women) (Table 3). Sarcomas which were not otherwise specified accounted for a considerable percentage of cases in both sexes (19% in men and 14% in women). The group of complex neoplasia was much more common in women than in men (sex ratio men/women 1:4.4) as carcinosarcomas including the Mullerian mixed tumour and endometrial stromal sarcomas are exclusively gynaecological tumours. The median age at diagnosis and the sex ratio differed considerably between the histological groups.

**Table 3:** Distribution of histological groups and selected histologies, year of diagnosis 2013

Diagnostic group	Men					Women					Sex ratio (M/F)
	Cases		Incidence rate per 100,000 per year		Age at diagnosis (Median)	Cases		Incidence rate per 100,000 per year		Age at diagnosis (Median)	
	N	%	Crude	Age-stand^a^		N	%	Crude	Age-stand^a^		
**Sarcoma, NOS**	**645**	**18.9**	**1.9**	**1.3**	**71.4**	**470**	**13.7**	**1.3**	**0.8**	**71.5**	**1.4**: **1**
Sarcoma, NOS	317	9.3	0.9	0.6	71.9	290	8.4	0.8	0.5	71.5	1.1: 1
Spindle cell sarcoma	72	2.1	0.2	0.1	73.5	53	1.5	0.1	0.1	73.4	1.4: 1
Giant cell sarcoma	170	5.0	0.5	0.3	72.9	87	2.5	0.2	0.1	72.5	2: 1
Small cell sarcoma	7	0.2	0.0	0.0	41.8	3	0.1	0.0	0.0	46.2	2.3: 1
Undifferentiated sarcoma	42	1.2	0.1	0.1	64.4	21	0.6	0.1	0.0	64.0	2: 1
**Fibrosarcoma**	**475**	**14.0**	**1.4**	**1.0**	**71.0**	**305**	**8.9**	**0.9**	**0.6**	**61.5**	**1.6**: **1**
Fibrosarcoma, NOS	48	1.4	0.1	0.1	64.0	36	1.0	0.1	0.1	67.3	1.3: 1
Fibromyxosarcoma	82	2.4	0.2	0.2	70.3	59	1.7	0.2	0.1	68.3	1.4: 1
Malignant fibrous histiocytoma	198	5.8	0.6	0.3	77.0	77	2.2	0.2	0.1	74.3	2.6: 1
Dermatofibrosarcoma, NOS	93	2.7	0.3	0.2	50.2	95	2.8	0.3	0.2	45.8	1: 1
**Liposarcoma**	**393**	**11.5**	**1.1**	**0.8**	**65.9**	**250**	**7.3**	**0.7**	**0.5**	**65.3**	**1.6**: **1**
Liposarcoma, NOS	144	4.2	0.4	0.3	70.2	111	3.2	0.3	0.2	68.5	1.3: 1
Liposarcoma, well differentiated	56	1.6	0.2	0.1	63.5	37	1.1	0.1	0.1	62.2	1.5: 1
Myxoid liposarcoma	69	2.0	0.2	0.2	56.5	41	1.2	0.1	0.1	52.8	1.7: 1
Dedifferentiated liposarcoma	89	2.6	0.3	0.2	70.8	32	0.9	0.1	0.1	69.6	2.8: 1
**Uterine leiomyosarcoma**	**0**	**0.0**	**0.0**	**0.0**	**.**	**109**	**3.2**	**0.3**	**0.2**	**60.4**	**–**
**Non-uterine leiomyosarcoma**	**277**	**8.1**	**0.8**	**0.6**	**70.5**	**330**	**9.6**	**0.9**	**0.6**	**67.3**	**1**: **1.2**
**Rhabdomyosarcoma**	**78**	**2.3**	**0.2**	**0.3**	**25.2**	**47**	**1.4**	**0.1**	**0.1**	**48.7**	**1.7**: **1**
Rhabdomyosarcoma, NOS	19	0.6	0.1	0.1	53.3	18	0.5	0.1	0.0	56.3	1.1: 1
Embryonal rhabdomyosarcoma	20	0.6	0.1	0.1	14.2	9	0.3	0.0	0.0	12.4	2.2: 1
**Complex Neoplasia**	**169**	**5.0**	**0.5**	**0.4**	**61.2**	**741**	**21.5**	**2.1**	**1.3**	**69.8**	**1**: **4.4**
Endometrial stromal sarcoma, NOS	0	0.0	0.0	0.0	.	75	2.2	0.2	0.1	62.2	–
Endometrial stromal sarcoma, low grade	0	0.0	0.0	0.0	.	36	1.0	0.1	0.1	53.3	–
Adenosarcoma	0	0.0	0.0	0.0	.	28	0.8	0.1	0.1	61.1	–
Mullerian mixed tumor	0	0.0	0.0	0.0	.	206	6.0	0.6	0.3	72.6	–
Carcinosarcoma, NOS	37	1.1	0.1	0.1	71.5	260	7.6	0.7	0.4	73.0	1: 7
Synovial sarcoma, NOS	32	0.9	0.1	0.1	56.2	28	0.8	0.1	0.1	49.5	1.1: 1
Synovial sarcoma, spindle cell	20	0.6	0.1	0.1	40.4	13	0.4	0.0	0.0	52.0	1.5: 1
**Phylloides tumour, malignant**	**0**	**0.0**	**0.0**	**0.0**	**.**	**56**	**1.6**	**0.2**	**0.1**	**56.2**	**–**
**Angiosarcoma**	**233**	**6.8**	**0.7**	**0.5**	**69.5**	**168**	**4.9**	**0.5**	**0.3**	**72.8**	**1.4**: **1**
Haemangiosarcoma, NOS	118	3.5	0.3	0.2	72.5	122	3.5	0.3	0.2	74.7	1: 1
Kaposi sarcoma	87	2.6	0.3	0.2	61.4	17	0.5	0.0	0.0	72.9	5.1: 1
**Osteosarcoma**	**85**	**2.5**	**0.2**	**0.3**	**35.8**	**67**	**1.9**	**0.2**	**0.2**	**39.8**	**1.3**: **1**
Osteosarcoma, NOS	69	2.0	0.2	0.2	42.0	49	1.4	0.1	0.1	45.3	1.4: 1
**Chondrosarcoma**	**105**	**3.1**	**0.3**	**0.2**	**60.8**	**119**	**3.5**	**0.3**	**0.2**	**61.4**	**1**: **1.1**
Chondrosarcoma, NOS	81	2.4	0.2	0.2	60.8	100	2.9	0.3	0.2	62.0	1: 1.2
**Giant cell neoplasia**	**4**	**0.1**	**0.0**	**0.0**	**56.6**	**5**	**0.1**	**0.0**	**0.0**	**58.3**	**1**: **1.3**
**Ewing family of tumours**	**107**	**3.1**	**0.3**	**0.4**	**24.7**	**70**	**2.0**	**0.2**	**0.2**	**25.1**	**1.5**: **1**
Ewing sarcoma	93	2.7	0.3	0.3	23.7	56	1.6	0.2	0.2	23.7	1.7: 1
**Malignant ameloblastoma**	**2**	**0.1**	**0.0**	**0.0**	**72.8**	**4**	**0.1**	**0.0**	**0.0**	**50.0**	**1**: **2**
**Chordoma**	**34**	**1.0**	**0.1**	**0.1**	**63.6**	**30**	**0.9**	**0.1**	**0.1**	**58.0**	**1.1**: **1**
Chordoma, NOS	30	0.9	0.1	0.1	64.5	27	0.8	0.1	0.1	58.0	1.1: 1
**Nerve sheath tumours**	**63**	**1.9**	**0.2**	**0.2**	**57.7**	**52**	**1.5**	**0.1**	**0.1**	**55.6**	**1.2**: **1**
Malignant peripheral nerve sheath tumour	56	1.6	0.2	0.1	57.6	43	1.2	0.1	0.1	56.0	1.3: 1
**GIST**	**734**	**21.6**	**2.1**	**1.5**	**69.5**	**619**	**18.0**	**1.7**	**1.1**	**70.2**	**1.2**: **1**

^a^age-standardized incidence rates per 100,000 (European standard 1976)

Bold text: Main groups

The distribution of histological groups depended on the site (see Additional file 3: Figure S1). While fibrosarcomas and angiosarcomas were the most predominant types in the head and neck, fibrosarcomas, liposarcomas and non-uterine leiomyosarcoma were most prominent in the limbs and trunk. In the thorax, 39% (men) and 36% (women) of sarcomas were not otherwise specified, and 22% (men) or 15% (women) had complex neoplasia. Angiosarcoma and fibrosarcoma were also common. In the abdomen, GIST represented 80% of sarcomas in both sexes. In the pelvis, distribution of histological groups differed considerably by sex. While in men, liposarcomas, non-uterine leiomyosarcomas, and rhabdomyosarcomas represent 64% of sarcoma cases, in women, complex neoplasia accounted for 71% of histologies. For bone sarcomas, about 34% were chondrosarcomas, about 26% osteosarcomas, and about 16% belonged to the Ewing family of tumours (data not shown).

**Fig. 2:**
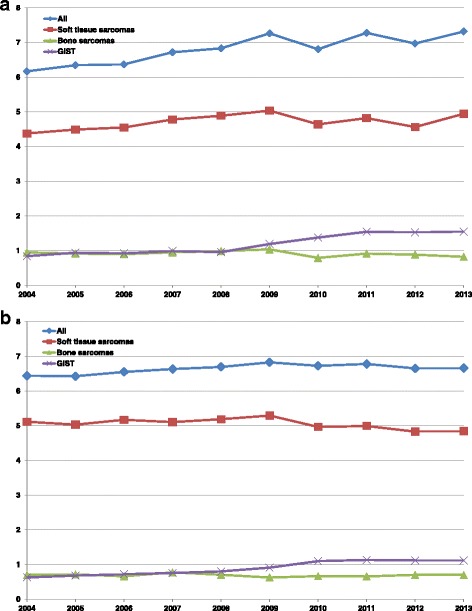
Trends of incidence rates for sarcoma entities, 2004–2013*. **a** Men; **b**Women. *per 100,000; age-standardized according to the European standard 1976, Hessen excluded, Nordrhein-Westfalen only Muenster region

Regarding the trend analysis for the years 2004 to 2013 (Fig. 2), there was a significant increase in incidence for men from 6.2 to 7.3 (APC 1.8% (95%-CI: 1.0 to 2.6)) and from 6.4 to 6.7 (AAPC 0.4% (95%-CI: 0.1 to 0.8)) for women (Table 4). It was due to a significant increase in incidence of GIST for both sexes: from 0.8 to 1.6 (APC 8.1% (95%-CI: 6.0 to 10.2)) in men and from 0.6 to 1.1 (APC 7.6% (95%-CI: 5.7 to 9.5)) in women. The incidence of soft tissue sarcomas (men) (APC 0.9% (95%-CI: -0.2 to 1.9)) as well as of bone sarcoma (both sexes, APC -1.1% (95%-CI: -3.1 to 0.9) for men and APC -0.5% (95%-CI: -2.0 to 1.1) for women) stayed constant over time (all incidence rates per 100.000, age-standardized according to European standard). As to soft tissue sarcoma in women, the incidence stayed constant up to the year 2009 and significantly decreased afterwards (one joinpoint detected). This lead to a decrease in the overall incidence of sarcoma after 2009 in women.

**Table 4:** Annual percentage change for incidence rates of sarcomas (all federal states except Baden-Wuerttemberg and Hessen; only Muenster for Nordrhein-Westfalen)

	Men					Women				
	Cases		Incidence Rate^a^		Change of Incidence Rate	Cases		Incidence Rate^a^		Change of Incidence Rate
	2004	2013	2004	2013	APC^b^ (%) [95% CI]	2004	2013	2004	2013	APC^b^(%) [95% CI]
All sarcoma	1752	2373	6.17	7.32	1.8 (1.0;2.6)*	2191	2463	6.44	6.66	0.4 (0.1;0.8)*,**
Soft tissue sarcoma	1256	1614	4.38	4.94	0.9 (−0.2;1.9)	1763	1816	5.11	4.85	−0.7 (−1.3;-0.1)*,**
Bone sarcoma	239	209	0.95	0.82	−1.1 (−3.1;0.9)	194	189	0.70	0.70	−0.5 (−2.0;1.1)
GIST	257	550	0.84	1.55	8.1 (6.0;10.2)*	234	458	0.63	1.12	7.6 (5.7;9.5)*

^a^age-standardized incidence rates per 100,000 (European standard 1976)

^b^APC = Annual Percentage Change

*statistically significant change (*p* < 0.05)

**one joinpoint detected, result for AAPC (Average annual percentage change)

The sarcoma incidence differed significantly between federal states in Germany (Table 5). For all sarcomas, it ranged between 5.5 (95%-CI: 5.0–6.1) (Sachsen-Anhalt) and 7.8 (95%-CI: 7.5–8.0 (Nordrhein-Westfalen) or 7.0–8.6 (Hamburg)) in men and between 5.2 (95%-CI: 4.7–5.8) (Sachsen-Anhalt) and 8.7 (95%-CI: 7.9–9.5) (Hamburg) in women (all incidence rates per 100,000, age-standardized). Within the sexes, the confidence interval of the federal state with the lowest incidence did not overlap with the one of the federal state with the highest incidence. Analyses for sarcoma subtypes were not sensible due to low case numbers in small federal states.

**Table 5:** Cases and age-standardized incidence^a^, by federal state, years of diagnosis 2009–2013, for all sarcomas

Federal State	All sarcoma			
	Men		Women	
	n	Incidence (95%-CI)^a^	n	Incidence (95%-CI)^a^
Bayern	2929	7.5 (7.3–7.8)	3253	7.3 (7.0–7.6)
Berlin	586	5.9 (5.4–6.4)	617	5.4 (5.0–5.9)
Brandenburg	545	6.6 (6.0–7.2)	643	6.7 (6.1–7.2)
Bremen	154	7.3 (6.1–8.5)	169	6.2 (5.1–7.2)
Hamburg	413	7.8 (7.0–8.6)	506	8.7 (7.9–9.5)
Hessen	1222	5.9 (5.6–6.3)	1276	5.5 (5.2–5.9)
Mecklenburg-Vorpommern	349	6.2 (5.5–6.9)	422	6.6 (5.9–7.3)
Niedersachsen	1943	7.6 (7.3–8.0)	1903	6.5 (6.2–6.8)
Nordrhein-Westfalen	4238	7.8 (7.5–8.0)	4467	7.2 (7.0–7.4)
Rheinland-Pfalz	916	6.9 (6.4–7.3)	926	6.2 (5.8–6.7)
Saarland	253	7.4 (6.4–8.3)	272	6.6 (5.7–7.5)
Sachsen	1080	7.6 (7.1–8.0)	1162	6.9 (6.4–7.3)
Sachsen-Anhalt	451	5.5 (5.0–6.1)	491	5.2 (4.7–5.8)
Schleswig-Holstein	612	6.6 (6.0–7.1)	725	7.0 (6.5–7.6)
Thueringen	474	6.3 (5.7–6.9)	544	6.2 (5.6–6.8)

*CI* Confidence interval

^a^age-standardized incidence rates per 100,000 (European standard 1976)

## Discussion

This study based on ten German population-based cancer registries and 6846 sarcoma cases diagnosed in 2013 examined the overall incidence of sarcomas in Germany and investigated whether there were statistically significant changes in incidence in the last 10 years. Analyses were based on the latest data available at present.

In general, the age-standardized incidence rates correspond well with the results from other studies [6–8, 10].

Trautmann et al. found higher age-adjusted (European standard) rates for **bone sarcomas** (2.6 per 100,000 for men and 1.8 for women) [9].

Regarding **GIST**, the age-standardized incidence rates (European standard) found in the literature were lower than in the presented study (0.1 to 0.9 per 100,000; men and women combined) [6, 8]. However, it was not explicitly stated whether GIST of unknown behaviour (ICD-O-3 8936/1), which represent a considerable proportion of all GIST, were included in analyses, as was done in our study. The significant increase in incidence for all sarcomas in the present study was due to a significant increase in incidence of GIST for both sexes as found in literature [14–16]. It can partly be explained by the increasing reliability of diagnosis due to the routine use of markers like CD 117 and DOG1 and because of the increasing awareness of the diagnosis by physicians [15, 16]. Before the third revision of the ICD-O classification (International Classification of Diseases for Oncology) [12] which has been introduced in 2003 in Germany, a separate code for GIST did not exist.

Excluding certain histologies according to expert opinion or because not being mentioned in the WHO classification 2002 did not considerably alter the results in men as they only accounted for a small number of cases. In women, after excluding Mullerian mixed tumours, the age-standardized incidence was lower than in men for soft tissue sarcomas. This is in accordance with other studies where they have been excluded as well [7–9].

The median age at diagnosis found in the present study was five to 10 years higher than in the literature for all entities [8, 16]. This may be partly due to the present study reporting more recent data with an ageing society in general. As well, in hospital-based studies [8], elderly patients who have not been treated in a hospital may have been missed.

The increasing incidence with age up to the ages of 80 to 84 and the decrease in the eldest age-group are in accordance with findings in the literature [8, 9, 14] and with most other cancer entities. It is usually explained by the lack of intensive diagnostic and treatment in elderly people. Therefore, these cases are neither reported to population-based cancer registries nor included in hospital-based studies. In accordance with the literature [8, 9], the age distribution for bone sarcomas in the study presented here revealed a second peak at ages 10 to 19. Hence, the median age at diagnosis of bone sarcomas was much lower than that of other sarcomas.

GIST, liposarcomas, leiomyosarcomas, and fibrosarcomas were the most important histological groups in the literature as well [5, 7, 8], although the respective proportions differed considerably between studies. In the literature, the complex neoplasia group was not categorized as an individual entity.

The median age at diagnosis and the sex ratio differed considerably between the histological groups. This is in accordance with histological and molecular genetic findings which show prominent differences between histologies [1, 3] and with other studies [5, 8].

The sarcoma incidence differed significantly between federal states in Germany. In cancer registration, it is hard to distinguish true differences in incidence rates from discrepancies due to differences in completeness or coding habits. As sarcoma is a rare cancer entity, numbers of cases were low for federal states with fewer inhabitants. Although incidence rates were calculated for a five-year period, the validity of these results is therefore restricted.

### Strengths and limitations

To our knowledge, this is the first study on sarcoma incidence in Germany based on high-quality data from population-based cancer registries. Analyses were based on the latest data available at present. The study has high external validity as it covered 87% of the German population. In addition, the high number of cases allowed for detailed stratified analyses, e.g. by individual histologies. Finally, the study included all sarcomas based on histology and not only sarcomas emerging from soft tissue.

A lack of completeness cannot be ruled out. However, there are only estimates for ICD-10 C41- to C49. As well, different coding habits between federal states may exist. Moreover, there is a high proportion of sarcomas not otherwise specified, which limits the analysis of histological subgroups. Finally, cases with unknown histologies were excluded. Thus, some sarcomas may have been missed. It has to be mentioned that all cases had been coded according to the WHO classification of 2002 but in 2013 a novel classification has been published. Furthermore, only local pathology reports were available and to date it is unclear if and in which cases reference pathology was performed which would increase the reliability of subtyping sarcomas.

## Conclusion

In summary, sarcoma incidence in Germany was examined based on data from population-based cancer registries for the first time. Detailed sub-analyses stratified by age, sex, and histology were performed. This study confirms the significant increase in incidence of GIST at the beginning of this century, which has also been found in literature. It can partly be explained by the increasing reliability of diagnosis due to the routine use of markers like CD 117 and DOG1 as well as by the increasing awareness of the diagnosis by physicians. The incidence of soft tissue sarcomas (only men) as well as of bone sarcoma stayed constant over time. In women, the incidence of soft tissue sarcomas significantly decreased after the year 2009.

## Additional files

Additional file 1: Table S1. Histologies not included in sensitivity analysis. Figure showing the number of cases with histologies not included in sensitivity analysis. (DOCX 15 kb)

Additional file 2: Table S2. Classification of sites according to ICD-O 3* Topography. Table showing the classification of sites according to ICD-O 3 (third edition of the International Classification of Diseases for Oncology) Topography. (DOCX 13 kb)

Additional file 3: Figure S1. Distribution of histological groups in different sites, year of diagnosis 2013. Figure showing the distribution of histological groups in different sites in Germany for the year of diagnosis 2013 via pie charts. (PDF 966 kb)

## Abbreviations

AAPC: Average annual percentage change
APC: Annual percentage change
CI: Confidence interval
GIST: Gastrointestinal stromal tumour
ICD: International Statistical Classification of Diseases and Related Health Problems
WHO: World Health Organization
ZfKD: German Centre for Cancer Registry Data
