# Supplementary material for: Strengthening health data on a rare and heterogeneous disease: sarcoma incidence and histological subtypes in Germany
Source: BMC Public Health. 2018 Feb 12;18:235. doi: 10.1186/s12889-018-5131-4 (PMC5809940; doi:10.1186/s12889-018-5131-4)
Supplement: Supplementary file 1 — Histologies not included in sensitivity analysis. Figure showing the number of cases with histologies not included in sensitivity analysis. (DOCX 15 kb) [file 12889_2018_5131_MOESM1_ESM.docx]

***Histologies not included in sensitivity analysis***

| ICD-O-3 M | Description | Reason for not including | Number of cases exluded, year of diagnosis 2013 | |
| --- | --- | --- | --- | --- |
|  |  |  | Men | Women |
| 8857 ** | Fibroblastic liposarcoma | Not in WHO-Classification | 1 | 0 |
| 8860* | Malignant angiomyolipoma | Expert opinion | 0 | 1 |
| 8861* | Malignant angiolipoma | Expert opinion | 0 | 0 |
| 8894 ** | Angiomyosarcoma | Not in WHO-Classification | 1 | 2 |
| 8895 | Myosarcoma | Not in WHO-Classification | 4 | 5 |
| 8902 | Mixed type rhabdomyosarcoma | Not in WHO-Classification | 1 | 0 |
| 8933 | Adenosarcoma | Expert opinion | 0 | 28 |
| 8940 | Malignant mixed tumor, NOS | Expert opinion | 6 | 4 |
| 8950 | Mullerian mixed tumor | Expert opinion | 0 | 206 |
| 8951 | Mesodermal mixed tumor | Expert opinion | 0 | 18 |
| 8959 | Malignant cystic nephroma | Expert opinion | 2 | 0 |
| 9020 | Phylloides tumor, malignant | Expert opinion | 0 | 56 |
| 9042 | Epitheloid Synovial Sarcoma | Not in WHO-Classification | 0 | 2 |
| 9133 | Epithelioid malignant hemangioendothelioma | Expert opinion | 8 | 12 |
| 9150** | Malignant hemangiopericytoma | Not in WHO-Classification | 14 | 8 |
| 9186 | Central osteosarcoma | Not in WHO-Classification | 0 | 3 |
| 9371 | Chondroid chordoma | Not in WHO-Classification | 4 | 2 |
| 9372 | Dedifferenciated chordoma | Not in WHO-Classification | 0 | 1 |
| **Total** | | | **41** | **348** |

* These histologies only exist as benign in the ICD-O-3 and WHO classification.

** These histologies only exist as benign or borderline behavior WHO classification.
