# Supplementary material for: Strengthening health data on a rare and heterogeneous disease: sarcoma incidence and histological subtypes in Germany
Source: BMC Public Health. 2018 Feb 12;18:235. doi: 10.1186/s12889-018-5131-4 (PMC5809940; doi:10.1186/s12889-018-5131-4)
Supplement: Supplementary file 2 — Classification of sites according to ICD-O 3* Topography. Table showing the classification of sites according to ICD-O 3 (third edition of the International Classification of Diseases for Oncology) Topography. (DOCX 13 kb) [file 12889_2018_5131_MOESM2_ESM.docx]

***Classification of sites according to ICD-O 3* Topography***

| Site | ICD-O 3* Topography | Description |
| --- | --- | --- |
| Head-Neck | C00-C14.9, C15.0, C32, C37, C70, C71, C72.2-C72.9, C73, C75.1, C75.3, C75.4, C44.0-4, C47.0, C49.0, C69, C30-C31, C76.0, C77.0 | Lip, oral cavity, pharynx, cervical oesophagus, larynx, thymus, meninges, brain, cranial nerves, thyroid gland, pituitary gland, pineal gland, carotid body, skin, peripheral nerves, connective tissue, lymph nodes of head and neck, eye, nasal cavity, middle ear, accessory sinuses, head, face or neck, nos |
| Limbs | C44.6, C44.7, C47.1, C47.2, C49.1, C49.2, C76.4, C76.5, C77.3, C77.4 | Skin, peripheral nerves, connective tissue, lymph nodes of limbs and limbs NOS |
| Trunk | C48.0, C50, C72.0, C72.1, C44.5, C47.3-C47.6, C49.3-C49.6, C76.1-C76.3, C76.7, C77.1, C77.2, C77.5 | Retroperitoneum, mamma, spinal cord, skin, peripheral nerves, connective tissue, lymph nodes of trunk, thorax, pelvis and abdomen |
| Thorax | C33,C34, C38,C39 | Trachea, lung, heart, mediastinum, pleura, other and ill-defined sites within respiratory system and intrathoracic organs |
| Abdomen | C15.1-C26, C42.2, C64.9, C74, C48.1, C48.2, C48.8 | Oesophagus, stomach, small intestine, colon, rectum, liver, gall bladder, biliary tract, pancreas, other and ill-defined digestive organs, spleen, kidney, adrenal gland, peritoneum |
| Pelvis | C51-C58, C60-C63, C65-C68 | Male and female genital organs, urinary organs except kidney |

* third edition of the International Classification of Diseases for Oncology
