# Supplementary material for: Strengthening health data on a rare and heterogeneous disease: sarcoma incidence and histological subtypes in Germany
Source: BMC Public Health. 2018 Feb 12;18:235. doi: 10.1186/s12889-018-5131-4 (PMC5809940; doi:10.1186/s12889-018-5131-4)
Supplement: Supplementary file 3 — Distribution of histological groups in different sites, year of diagnosis 2013. Figure showing the distribution of histological groups in different sites in Germany for the year of diagnosis 2013 via pie charts. (PDF 966 kb) [file 12889_2018_5131_MOESM3_ESM.pdf]

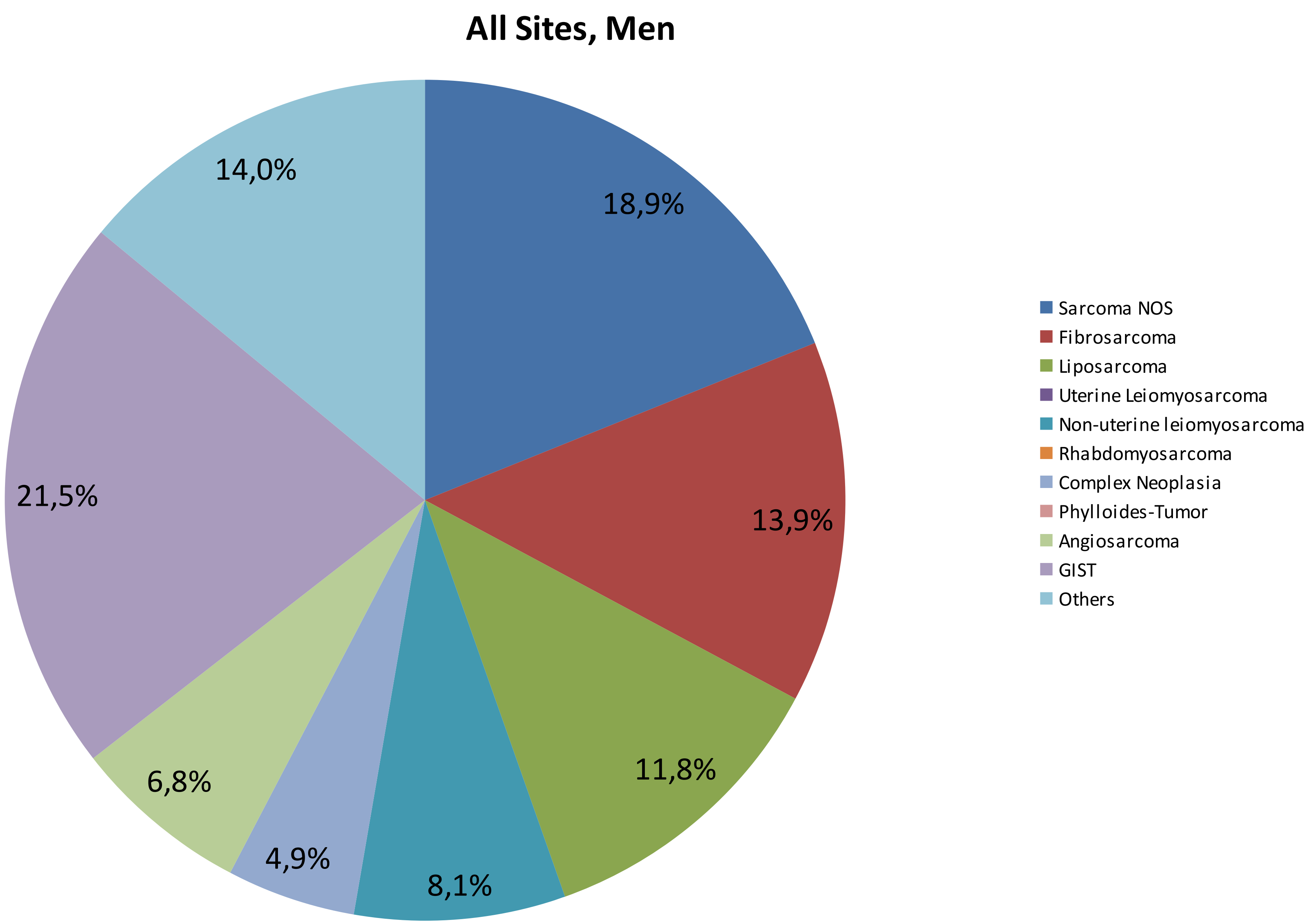
All Sites, Men
18,9%
13,9%
11,8%
8,1%
4,9%
6,8%
21,5%
14,0%
Sarcoma NOS
Fibrosarcoma
Liposarcoma
Uterine Leiomyosarcoma
Non-uterine leiomyosarcoma
Rhabdomyosarcoma
Complex Neoplasia
Phylloides-Tumor
Angiosarcoma
GIST
Others

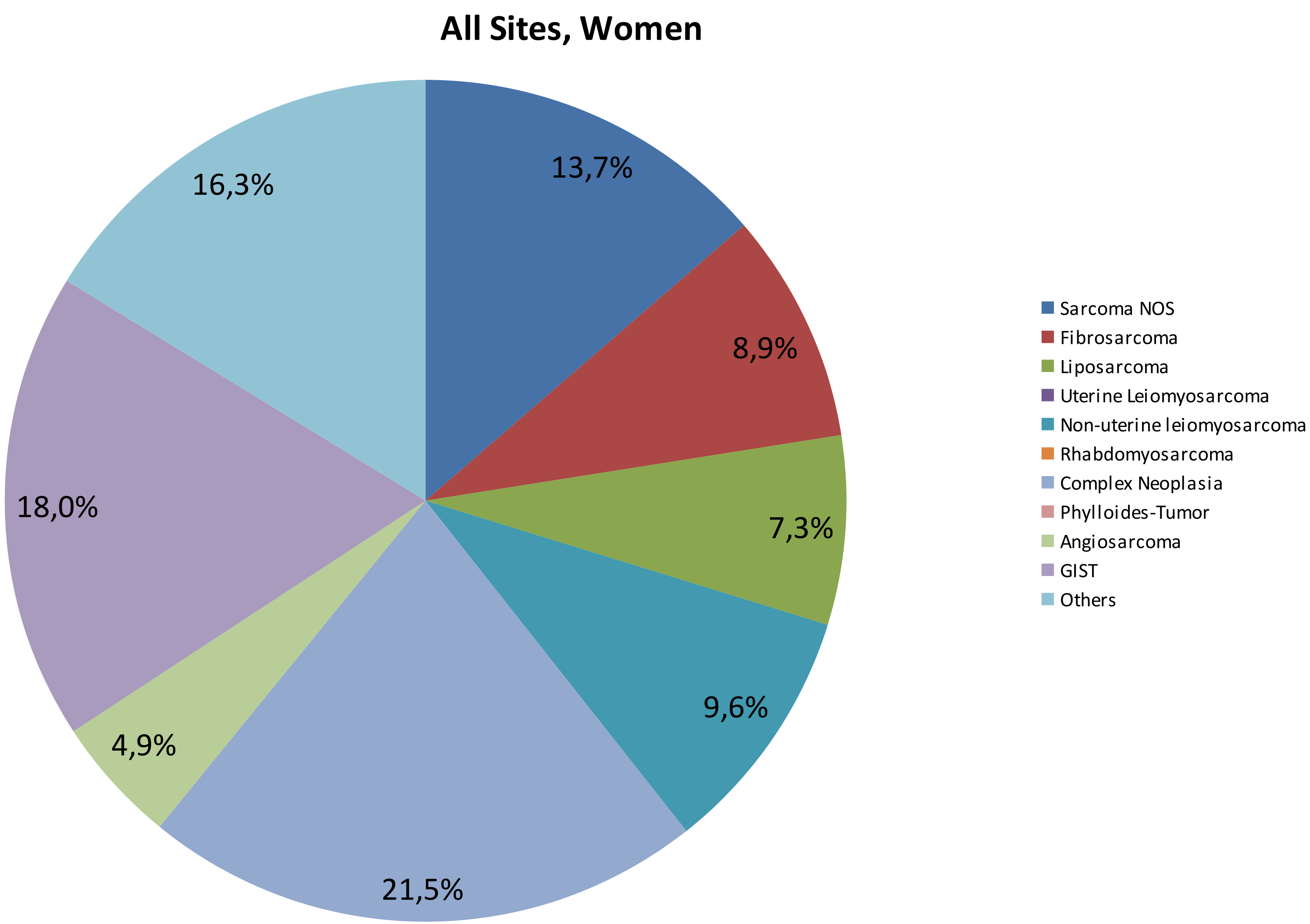

All Sites, Women
13,7%
8,9%
7,3%
9,6%
21,5%
4,9%
18,0%
16,3%
Sarcoma NOS
Fibrosarcoma
Liposarcoma
Uterine Leiomyosarcoma
Non-uterine leiomyosarcoma
Rhabdomyosarcoma
Complex Neoplasia
Phylloides-Tumor
Angiosarcoma
GIST
Others

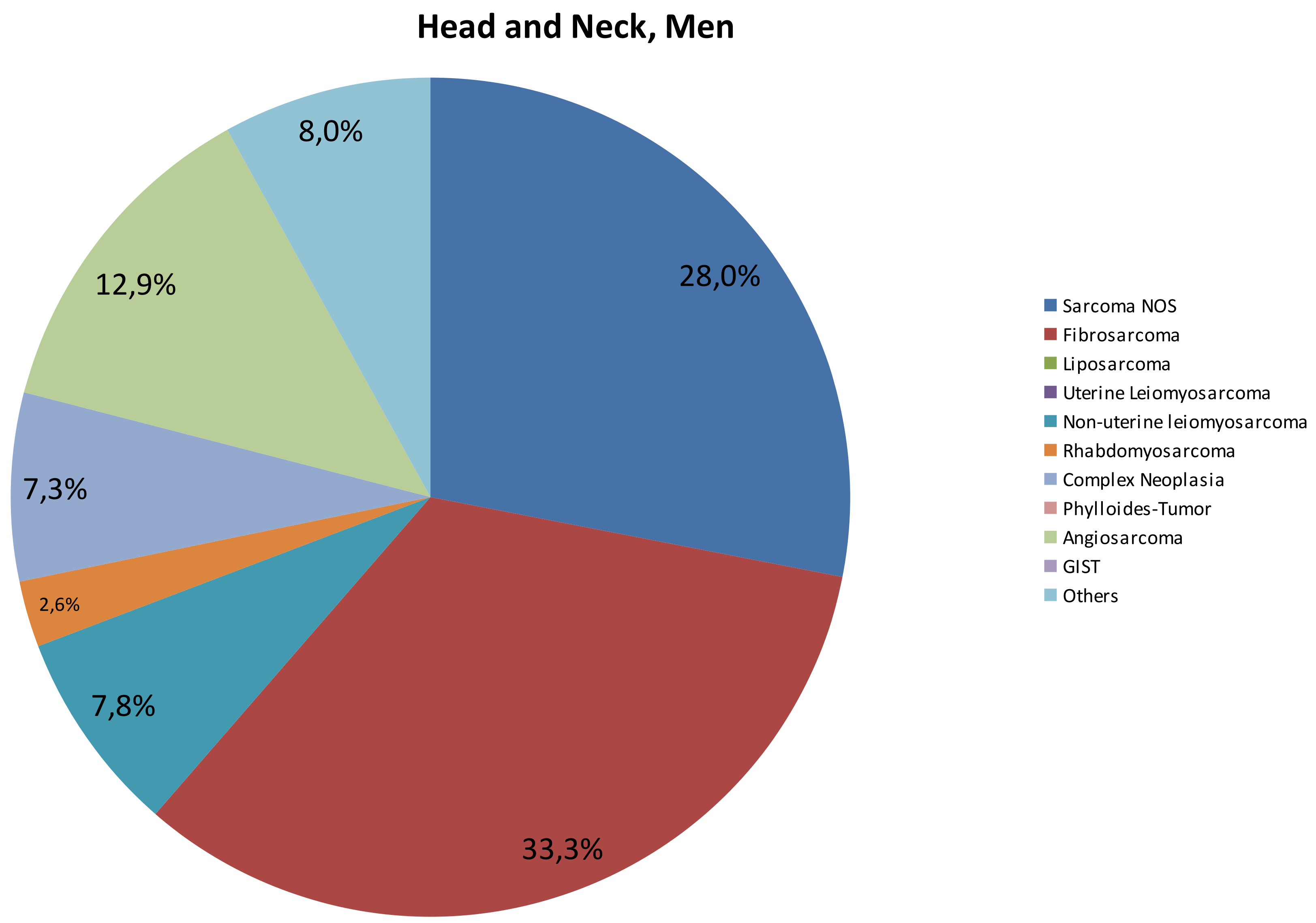
Head and Neck, Men
28,0%
33,3%
7,8%
2,6%
7,3%
12,9%
8,0%
Sarcoma NOS
Fibrosarcoma
Liposarcoma
Uterine Leiomyosarcoma
Non-uterine leiomyosarcoma
Rhabdomyosarcoma
Complex Neoplasia
Phylloides-Tumor
Angiosarcoma
GIST
Others

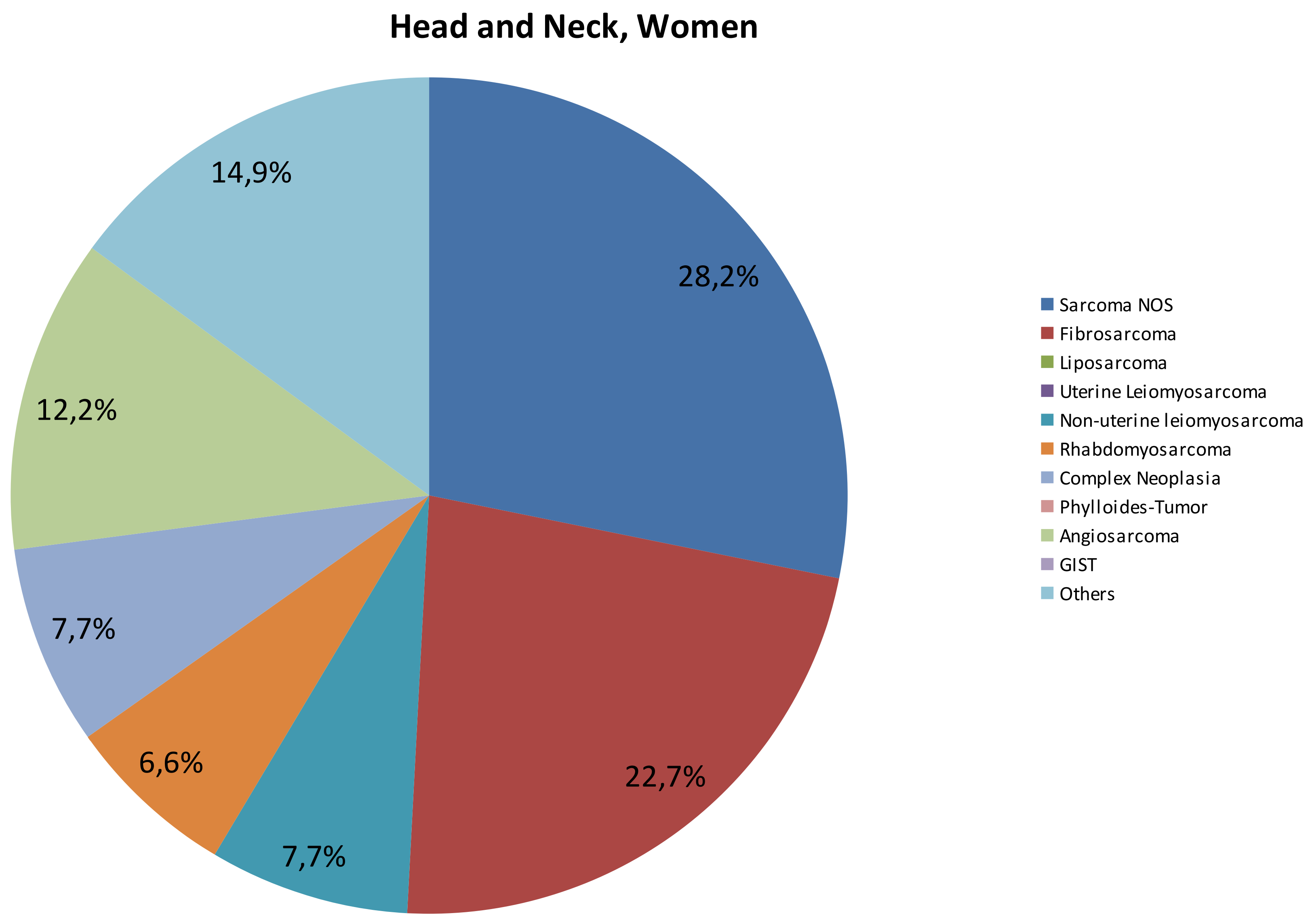

Head and Neck, Women
28,2%
22,7%
7,7%
6,6%
7,7%
12,2%
14,9%
Sarcoma NOS
Fibrosarcoma
Liposarcoma
Uterine Leiomyosarcoma
Non-uterine leiomyosarcoma
Rhabdomyosarcoma
Complex Neoplasia
Phylloides-Tumor
Angiosarcoma
GIST
Others

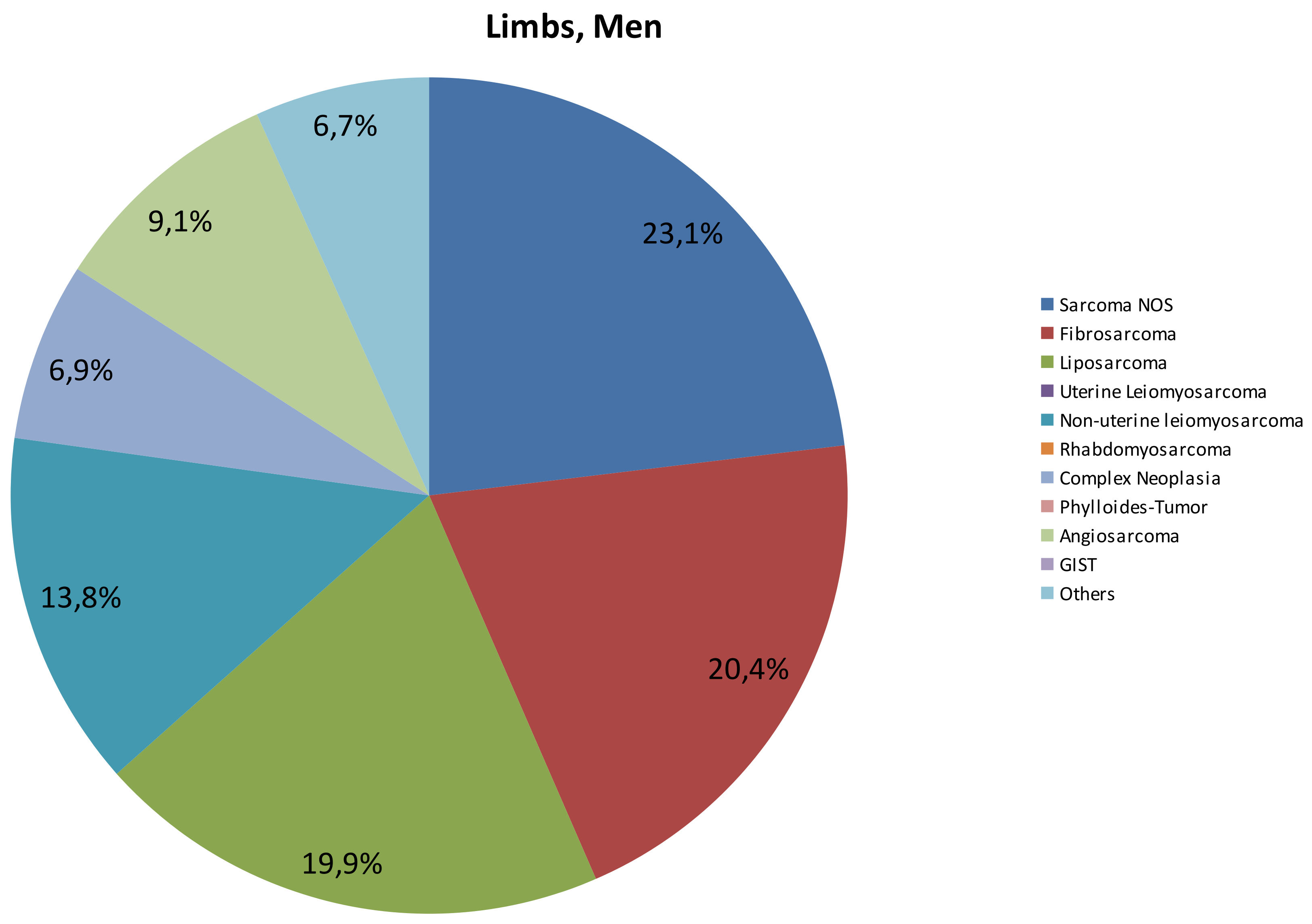
Limbs, Men
23,1%
20,4%
19,9%
13,8%
6,9%
9,1%
6,7%
Sarcoma NOS
Fibrosarcoma
Liposarcoma
Uterine Leiomyosarcoma
Non-uterine leiomyosarcoma
Rhabdomyosarcoma
Complex Neoplasia
Phylloides-Tumor
Angiosarcoma
GIST
Others

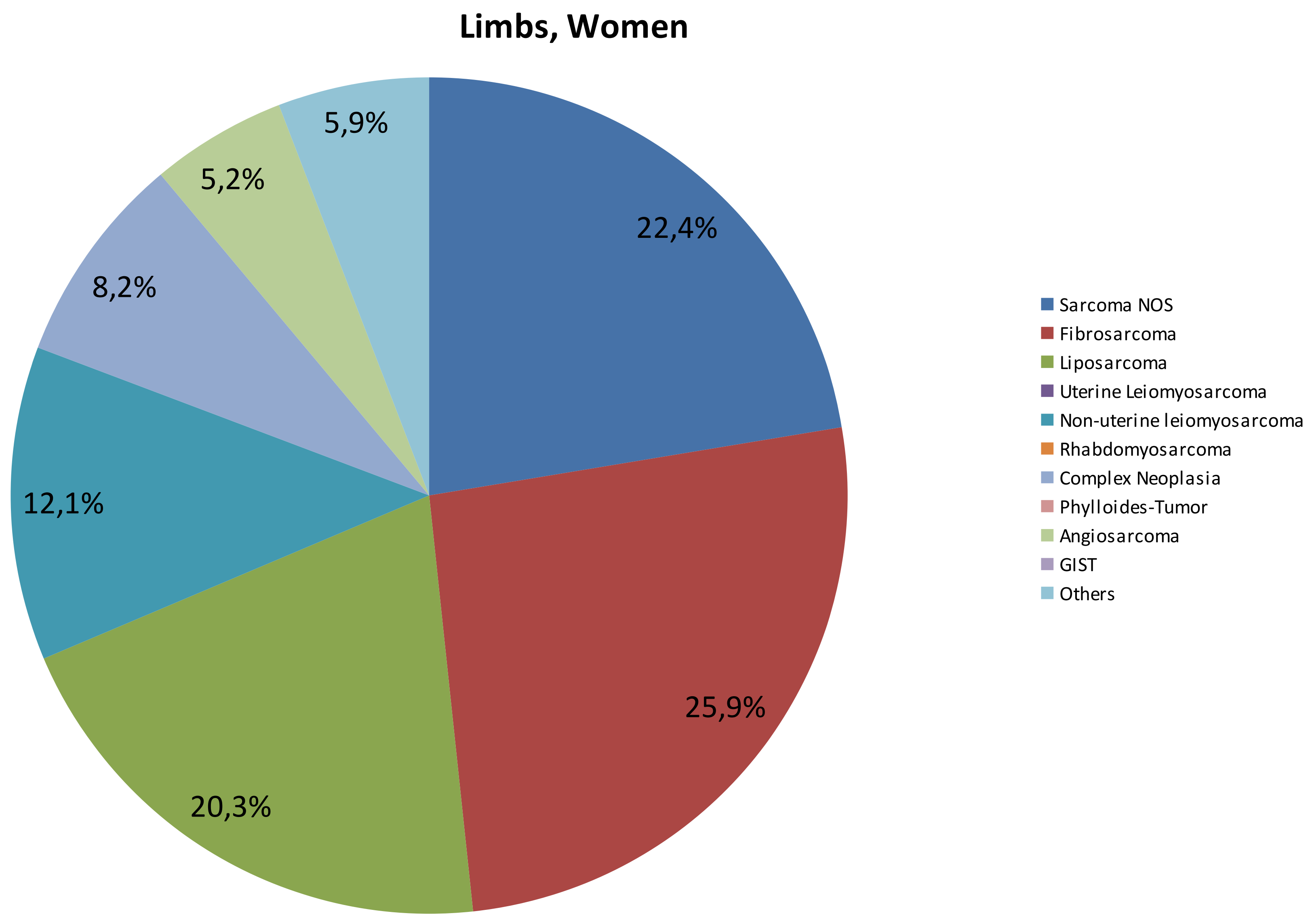

Limbs, Women
22,4%
25,9%
20,3%
12,1%
8,2%
5,2%
5,9%
Sarcoma NOS
Fibrosarcoma
Liposarcoma
Uterine Leiomyosarcoma
Non-uterine leiomyosarcoma
Rhabdomyosarcoma
Complex Neoplasia
Phylloides-Tumor
Angiosarcoma
GIST
Others

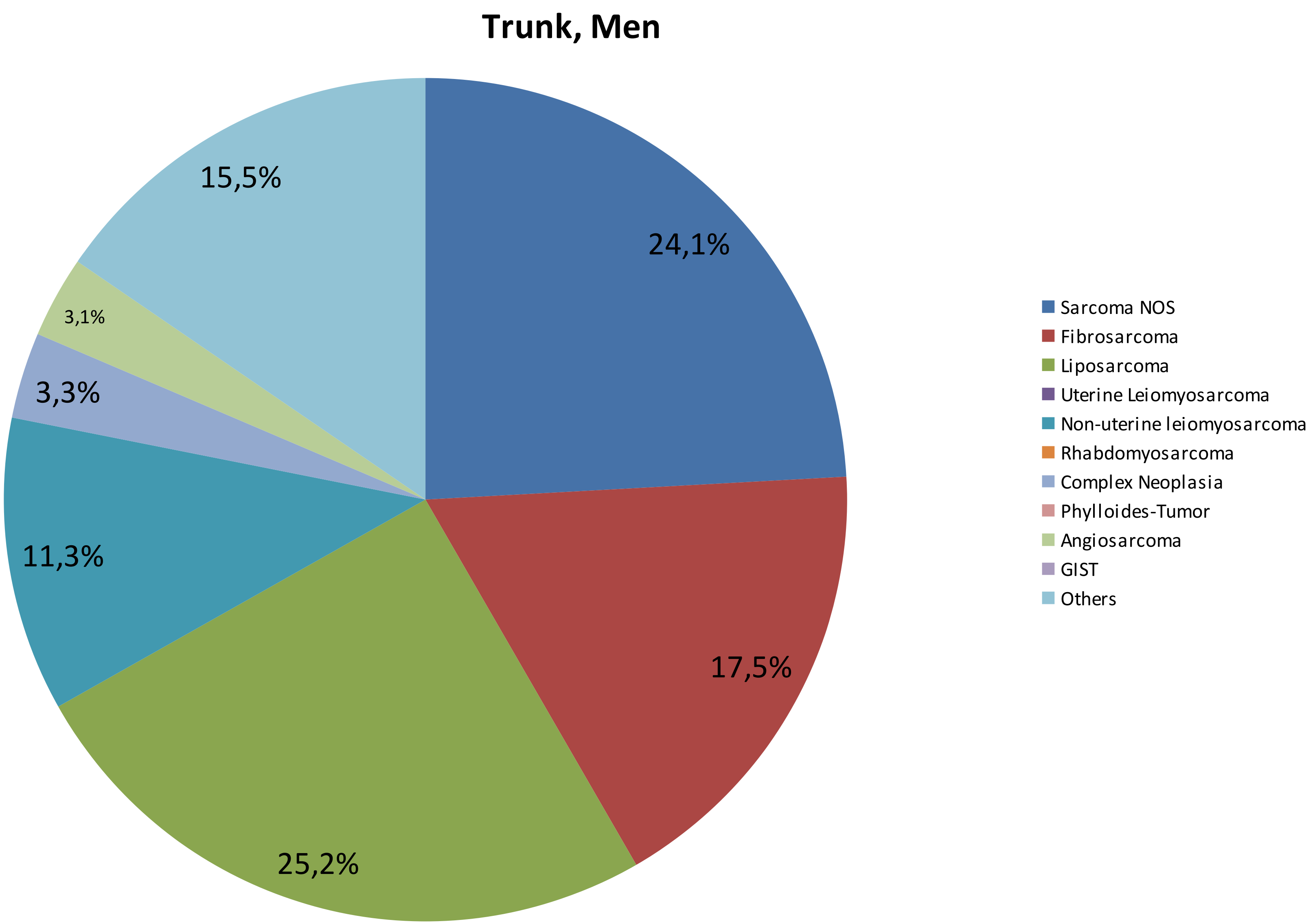

Trunk, Men
24,1%
17,5%
25,2%
11,3%
3,3%
3,1%
15,5%
Sarcoma NOS
Fibrosarcoma
Liposarcoma
Uterine Leiomyosarcoma
Non-uterine leiomyosarcoma
Rhabdomyosarcoma
Complex Neoplasia
Phylloides-Tumor
Angiosarcoma
GIST
Others

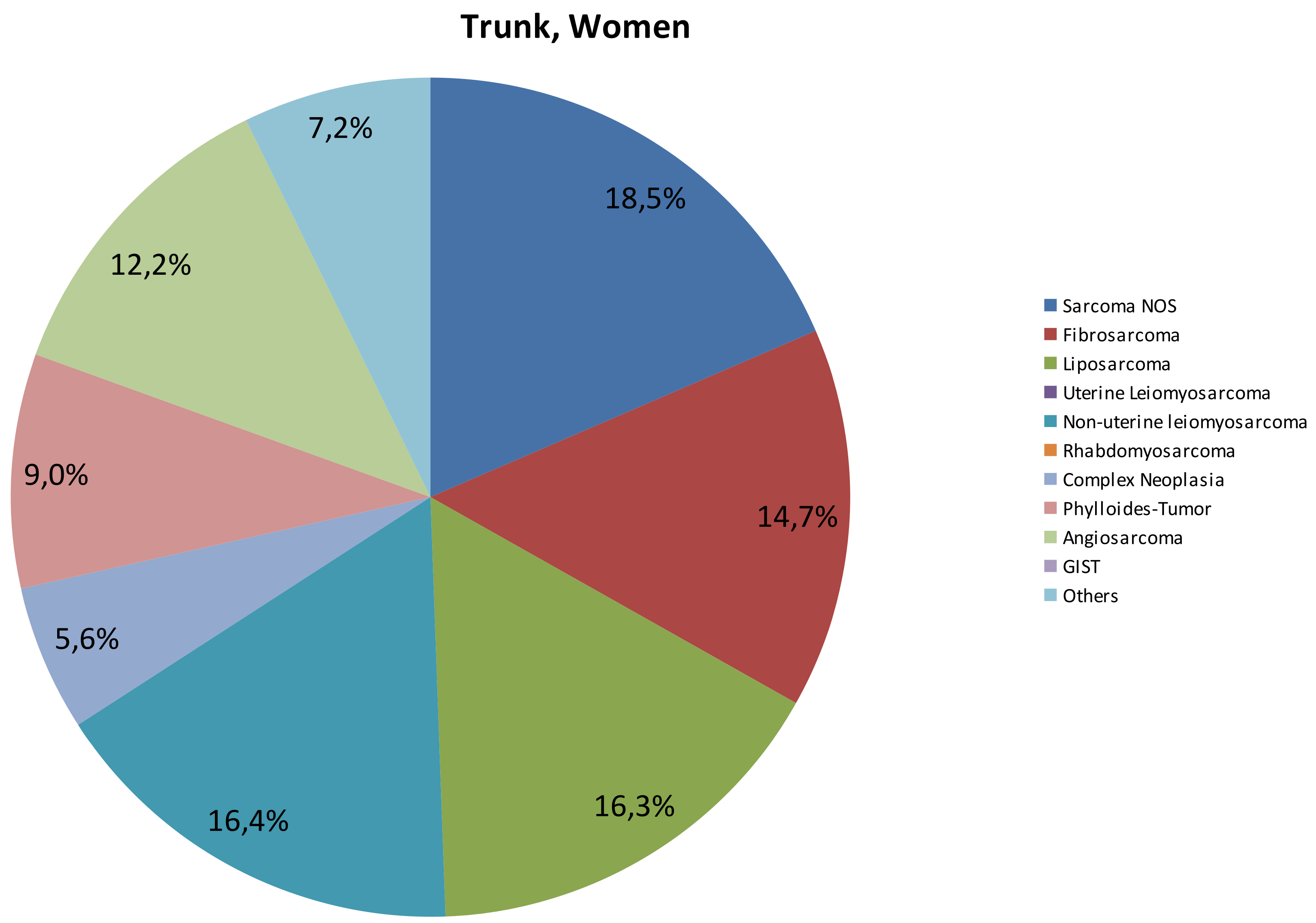
Trunk, Women
18,5%
14,7%
16,3%
16,4%
5,6%
9,0%
12,2%
7,2%
Sarcoma NOS
Fibrosarcoma
Liposarcoma
Uterine Leiomyosarcoma
Non-uterine leiomyosarcoma
Rhabdomyosarcoma
Complex Neoplasia
Phylloides-Tumor
Angiosarcoma
GIST
Others

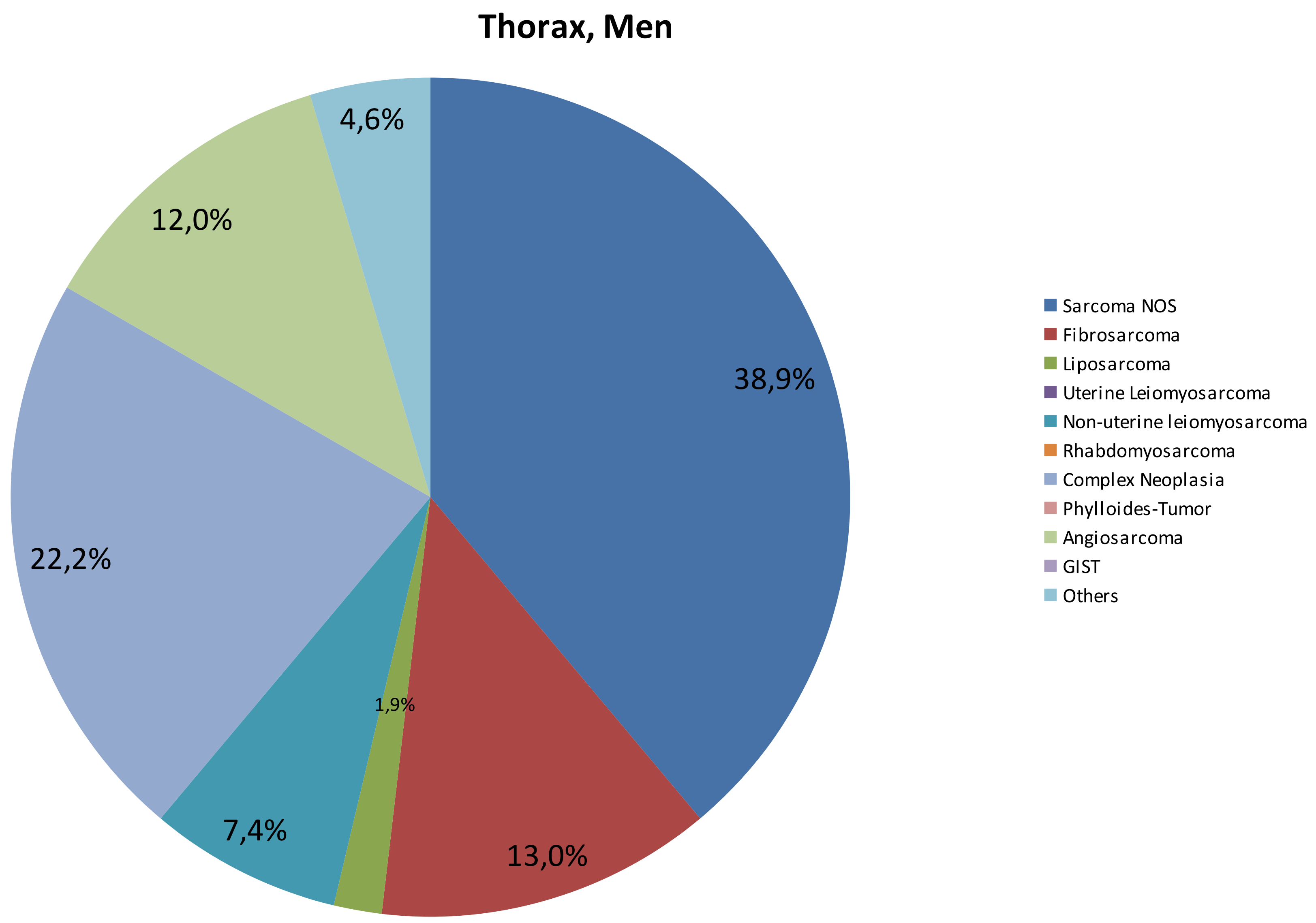

Thorax, Men
38,9%
13,0%
1,9%
7,4%
22,2%
12,0%
4,6%
Sarcoma NOS
Fibrosarcoma
Liposarcoma
Uterine Leiomyosarcoma
Non-uterine leiomyosarcoma
Rhabdomyosarcoma
Complex Neoplasia
Phylloides-Tumor
Angiosarcoma
GIST
Others

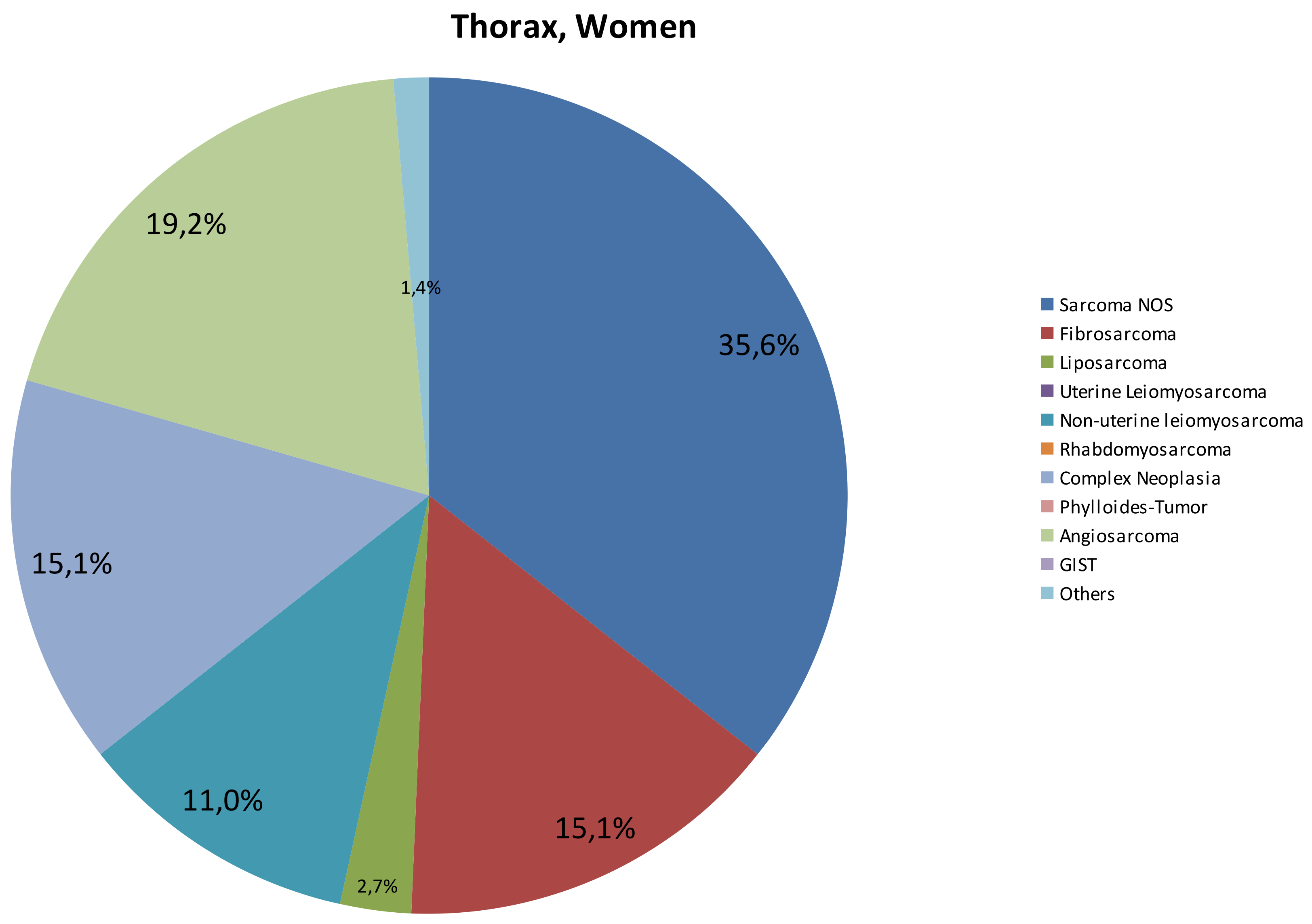
Thorax, Women
35,6%
15,1%
2,7%
11,0%
15,1%
19,2%
1,4%
Sarcoma NOS
Fibrosarcoma
Liposarcoma
Uterine Leiomyosarcoma
Non-uterine leiomyosarcoma
Rhabdomyosarcoma
Complex Neoplasia
Phylloides-Tumor
Angiosarcoma
GIST
Others

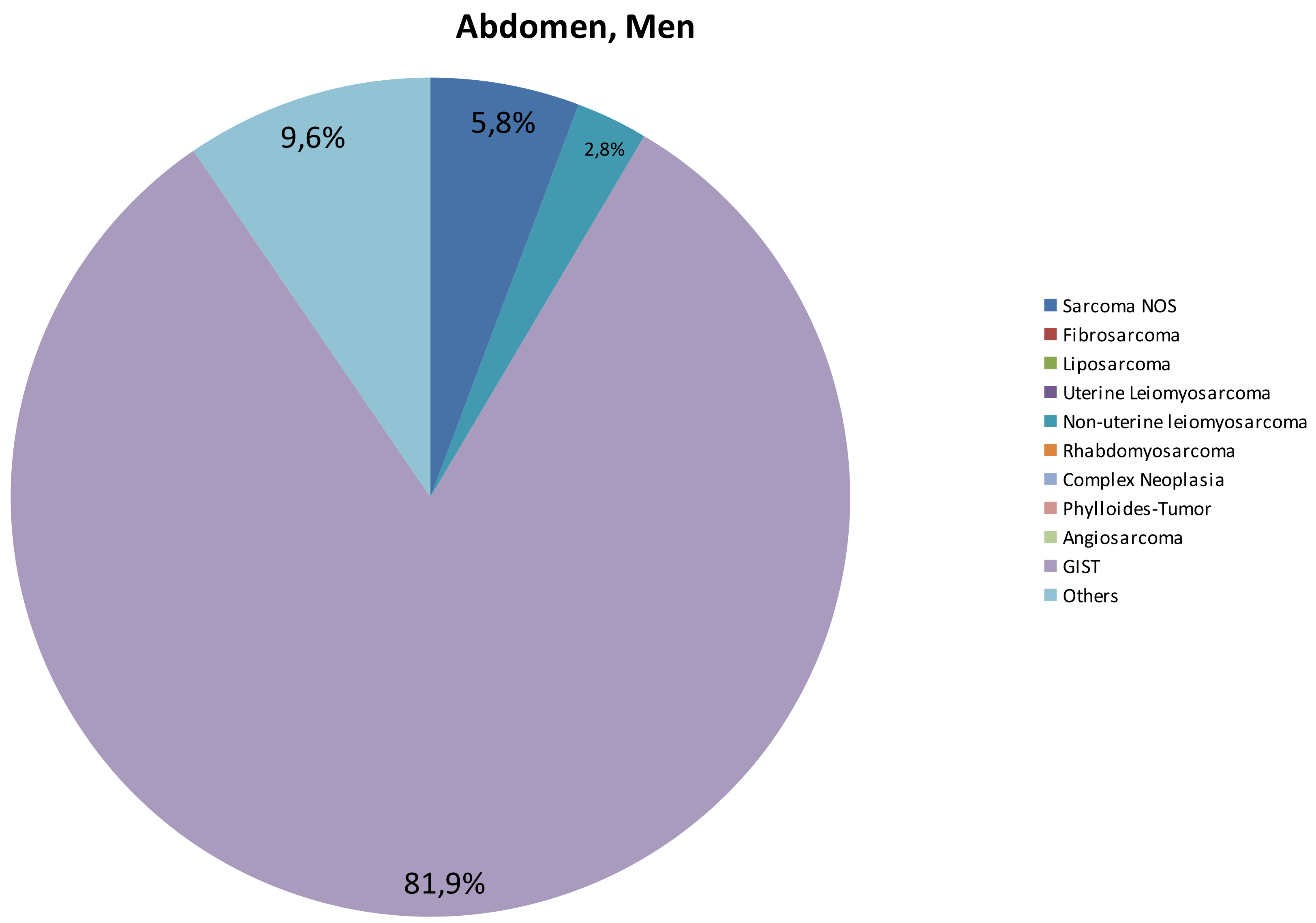
Abdomen, Men
5,8%
2,8%
9,6%
81,9%
Sarcoma NOS
Fibrosarcoma
Liposarcoma
Uterine Leiomyosarcoma
Non-uterine leiomyosarcoma
Rhabdomyosarcoma
Complex Neoplasia
Phylloides-Tumor
Angiosarcoma
GIST
Others

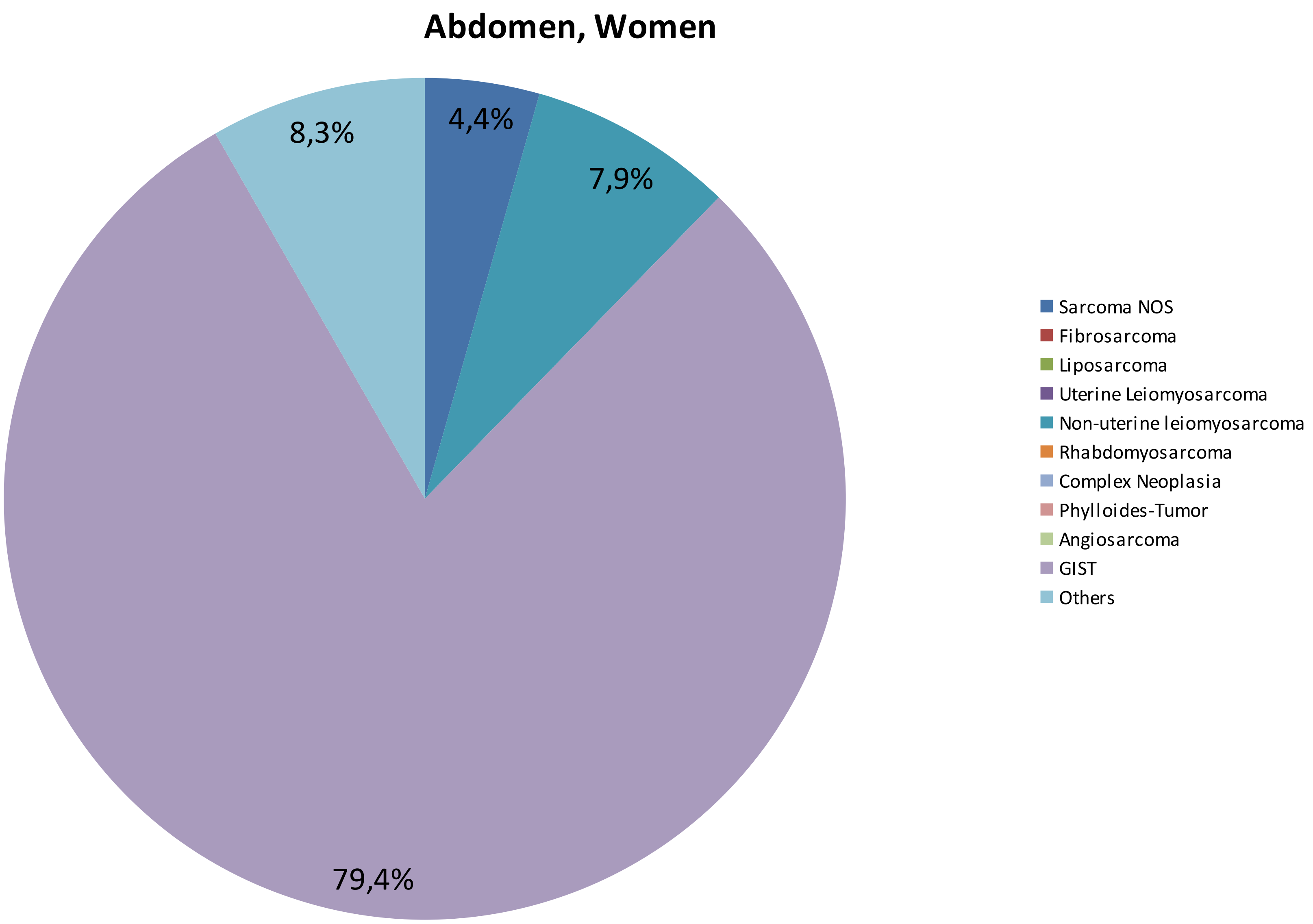
Abdomen, Women
4,4%
7,9%
8,3%
79,4%
Sarcoma NOS
Fibrosarcoma
Liposarcoma
Uterine Leiomyosarcoma
Non-uterine leiomyosarcoma
Rhabdomyosarcoma
Complex Neoplasia
Phylloides-Tumor
Angiosarcoma
GIST
Others

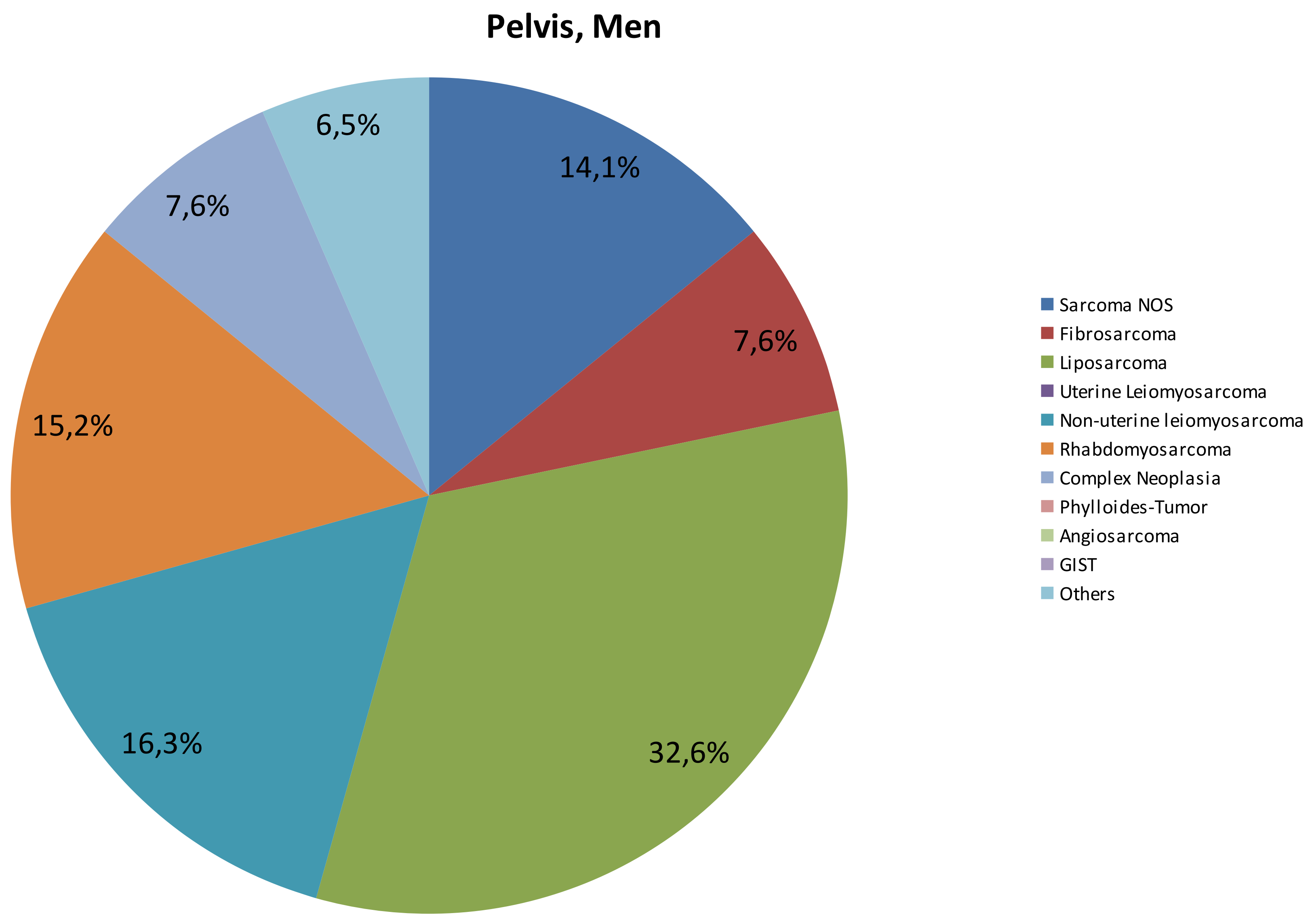
Pelvis, Men
6,5%
14,1%
7,6%
7,6%
15,2%
16,3%
32,6%
Sarcoma NOS
Fibrosarcoma
Liposarcoma
Uterine Leiomyosarcoma
Non-uterine leiomyosarcoma
Rhabdomyosarcoma
Complex Neoplasia
Phylloides-Tumor
Angiosarcoma
GIST
Others

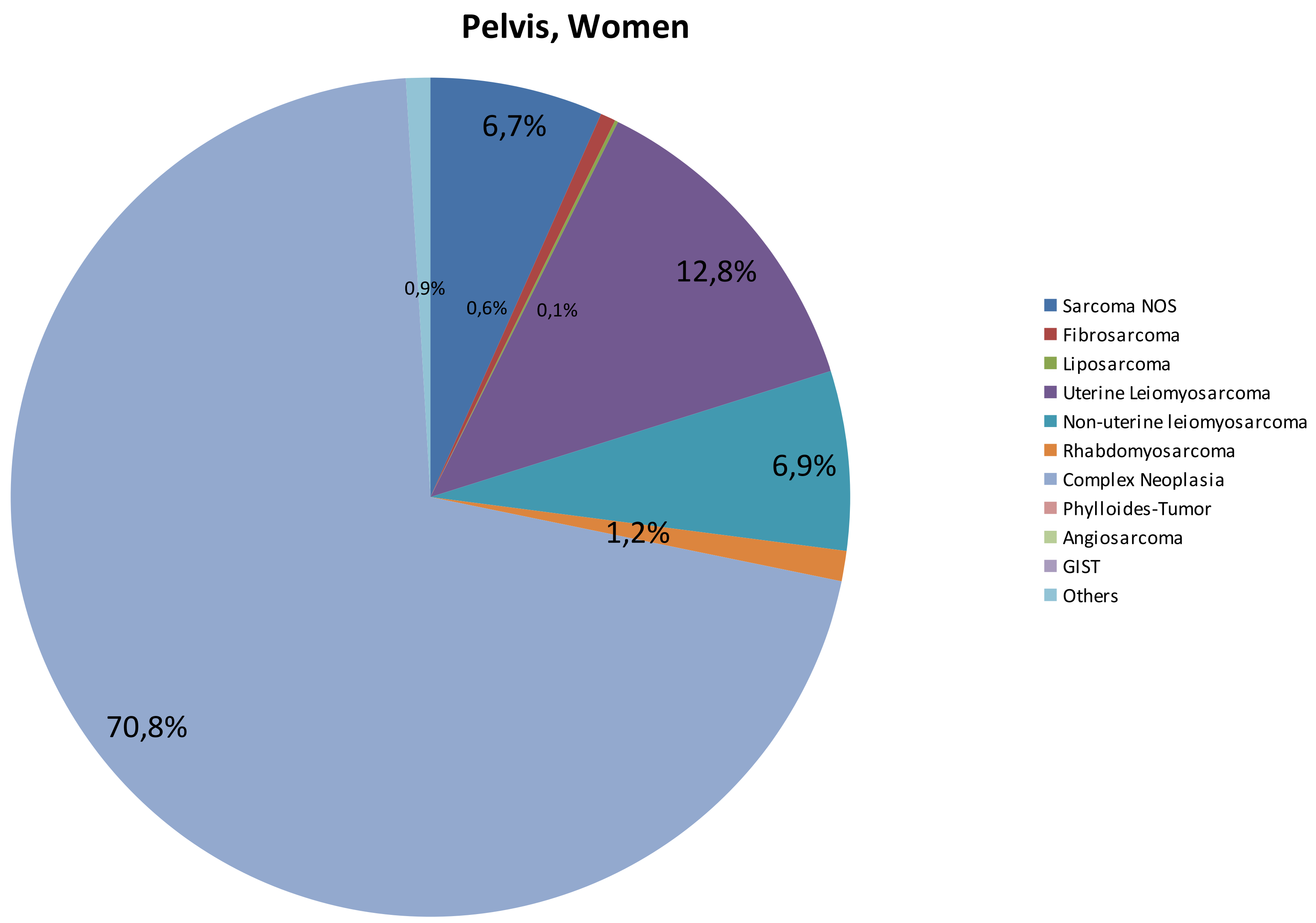
Pelvis, Women
6,7%
0,9%
0,6%
0,1%
12,8%
6,9%
1,2%
70,8%
Sarcoma NOS
Fibrosarcoma
Liposarcoma
Uterine Leiomyosarcoma
Non-uterine leiomyosarcoma
Rhabdomyosarcoma
Complex Neoplasia
Phylloides-Tumor
Angiosarcoma
GIST
Others
